# 2068: Expanding capacity for Clinical and Translational Science by investing in research staff through the strategic teamwork for effective practice-mentor development program (STEP-MDP)

**DOI:** 10.1017/cts.2017.157

**Published:** 2018-05-10

**Authors:** Christine Marie Denicola, Lisa Altshuler, Sondra Zabar

**Affiliations:** General Clinical Research Center, New York University, New York, NY, USA

## Abstract

OBJECTIVES/SPECIFIC AIMS: Skillful research staff members are critical to productive translational research teams and yet their ongoing professional development is rarely formally addressed. Through the Strategic Teamwork for Effective Practice-Mentor Development Program (STEP-MDP), we aimed to both create a community of practice (COP) for research staff and build the skills needed to enhance research team performance. METHODS/STUDY POPULATION: We selected 16 participants of 32 staff-level applicants from among the NYU Schools of Medicine, Social Work and Nursing for the first STEP-MDP cohort. Participants included research assistants, coordinators, managers, and directors. We delivered 3, two-hour workshops, scheduled 3 weeks apart, focused on team communication, identifying team areas for improvement, and mentorship/coaching skills. Peer-Coaching Teams (PCTs) were created by pairing participants at the same position level, and PCTs worked together at each session to explore and practice learned skills. Sessions featured brief didactics, group-based learning and exercises based on participants’ real issues. A variety of active learning techniques such as brainstorming, role-playing, problem solving, and peer coaching were used. Practical core readings, worksheets, and summary cards were provided. PCTs met between sessions to practice coaching skills, and troubleshoot problems. RESULTS/ANTICIPATED RESULTS: Participants (n=16) completed a 37-item retrospective pre/post self-assessment of team behaviors and skills, and a STEP-MDP evaluation survey at the end. We saw pre-post improvements in each of 5 self-assessment domains: Communication (4 items, pre-mean 2.66, post mean 3.36, *p*≤0.001), Leadership (8 items, pre-mean 2.76, post mean 3.55, *p*≤0.001), Empowerment and Motivation (12 items, pre-mean 2.86, post mean 3.51, *p*≤0.001), Coaching (6 items, pre-mean 2.40, post mean 3.58, *p*≤0.001), and Community (3 items, pre-mean 2.33, post mean 3.76, *p*≤0.001). On average, PCTs met twice (range 2–4 times) between workshop sessions. Learners valued the PCTs, and 1 commented on the value of working with peers in PCTs, having no one in a similar position within his immediate work environment. Participants’ written comments strongly endorsed the value of the workshops for their work, with the coaching skills session seen as the most valuable. Some participants worry that skills will decrease over time without continued reinforcement. All but 1 participant reported that they planned to continue with the PCT. DISCUSSION/SIGNIFICANCE OF IMPACT: The number of applicants to our program suggests a need and motivation for staff to participate in the STEP-MDP. Participants’ reported improved skills and sense of community. To maintain the COP and address worry about degradation of skills we are planning to remind PCTs to meet once a month and will follow-up with them 3 and 6 months post intervention to evaluate their continued development. This spring a second cohort will receive the training. We believe developing these core teamwork skills will lead to more collaborative, efficient, and innovative research. We have implemented a successful program targeting critical members of research teams with potential to facilitate expansion of institutional capacity for translational research. It will be important to understand the long-term impact of the program on individuals, on team science, on research, and ultimately on the health of the public.

